# Prognostic value of HPV-mRNA in sentinel lymph nodes of cervical cancer patients with pN0-status

**DOI:** 10.18632/oncotarget.4132

**Published:** 2015-05-14

**Authors:** Matthias Dürst, Heike Hoyer, Christoph Altgassen, Christiane Greinke, Norman Häfner, Alba Fishta, Mieczyslaw Gajda, Ute Mahnert, Peter Hillemanns, Thomas Dimpfl, Miriam Lenhard, K. Ulrich Petry, Ingo B. Runnebaum, Achim Schneider

**Affiliations:** ^1^ Department of Gynecology, Jena University Hospital, Friedrich-Schiller-University, Jena, Germany; ^2^ Institute of Medical Statistics, Information Sciences and Documentation, Jena University Hospital, Friedrich-Schiller-University, Jena, Germany; ^3^ Department of Gynecology and Obstetrics, Universitätsklinikum Schleswig-Holstein, Lübeck, Germany; ^4^ Institute of Pathology, Jena University Hospital, Friedrich-Schiller-University, Jena, Germany; ^5^ Department of Gynecology and Obstetrics, Helios Klinikum Erfurt GmbH, Erfurt, Germany; ^6^ Department of Gynecology and Obstetrics, Medizinische Hochschule Hannover, Hannover, Germany; ^7^ Department of Gynecology and Obstetrics, Klinikum Kassel GmbH, Kassel, Germany; ^8^ Department of Gynecology and Obstetrics, Ludwig-Maximilians-University Munich, Campus Grosshadern, Munich, Germany; ^9^ Department of Gynecology and Obstetrics, Klinikum Wolfsburg, Wolfsburg, Germany; ^10^ Institute for Cytology and Dysplasia, Fürstenbergkarree, Berlin, Germany

**Keywords:** sentinel lymph node, cervical cancer, HPV-mRNA, prognosis, disease free survival

## Abstract

Up to 15% of patients with cervical cancer and pN0-status develop recurrent-disease. This may be due to occult metastatic spread of tumor cells. We evaluated the use of human-papillomavirus-(HPV)-mRNA as a molecular marker for disseminated tumor cells to predict the risk of recurrence. For this prospective, multi-center prognostic study, 189 patients free of lymphnode metastases by conventional histopathology could be analyzed. All patients underwent complete lymphadenectomy. Of each sentinel node (SLN) a biopsy was taken for the detection of HPV-E6-E7-mRNA. Median follow-up time after surgery was 8.1 years. HPV-mRNA could be detected in SLN of 52 patients (27.5%). Recurrence was observed in 22 patients. Recurrence-free-survival was significantly longer for patients with HPV-negative SLN (log rank *p* = 0.002). By Cox regression analysis the hazard ratio (95%CI) for disease-recurrence was 3.8 (1.5 – 9.3, *p* = 0.004) for HPV-mRNA-positive compared to HPV-mRNA-negative patients. After adjustment for tumor size as the most influential covariate the HR was still 2.8 (1.1 – 7.0, *p* = 0.030). In patients with cervical cancer and tumor-free lymph nodes by conventional histopathology HPV-mRNA-positive SLN were of prognostic value independent of tumor size. Particularly, patients with tumors larger than 20mm diameter could possibly benefit from further risk stratification using HPV-mRNA as a molecular marker.

## INTRODUCTION

In patients with cervical cancer, metastatic spread to lymph nodes is known to be the most important prognostic parameter which is crucial for selection of therapy. The risk of lymph node metastases in women with early stage cervical cancer (up to FIGO stage IB1) is approximately 15% and as a consequence the majority of patients do not benefit from lymphadenectomy [1, 2]. Radical lymphadenectomy leads to lymphedema, serocele formation, paresthesia, voiding disorders, and possibly reduced immune competence. In recent years the use of sentinel lymph node biopsy (SLN) was explored extensively. The concept is based on the assumption that if the first draining lymph node is free of disease, all other lymph nodes in the nodal basin should also be free of disease [3]. An initial study examining the utility of SLN biopsy in patients diagnosed with cervical cancer showed great promise with a detection rate, sensitivity and negative predictive value of 94%, 90.9% and 99.1%, respectively for patients with tumors measuring 2cm or less in diameter [4]. In more recent studies it was shown that sensitivity was close to 100% in patients where SLNs were detected bilaterally [5-7]. Moreover, the detection of nodal metastasis was improved by ultrastaging which comprises multiple serial sectioning and immunohistochemical staining of the SLN [6, 7]. Indeed, there is even a report that sentinel lymph node biopsy in early stage cancer is a more sensitive procedure in detecting pelvic lymph node metastases compared to complete lymphadenectomy [8]. Ultrastaging allows the detection of low-volume disease which includes micrometastasis (MM) and isolated tumor cells (ITC). By definition, macrometastasis are tumor deposits >2mm, micrometastasis are deposits between >0.2 and 2mm, and ITC are deposits ≤0.2mm including the presence of single non-cohesive cytokeratin-positive tumor cells [9]. However, the prognostic implications of low volume metastatic disease (micrometastasis and ITC) for patients with cervical cancer are not yet fully understood and serve only partly as prognostic indicators [6, 10].

The identification of ITC, in particular single tumor cells, requires a highly specific marker. In this context, the detection of cytokeratins by immunohistochemical staining or at the mRNA level is not ideal [11, 12]. Moreover, in a recent study it was shown that benign epithelial cells can be displaced iatrogenetically and transported to sentinel lymph nodes [13]. Thus, in order to address the clinical significance of small numbers of tumor cells in SLN, it is mandatory to use the most specific markers available. Characteristic of all cervical cancer cells is the presence of high-risk-(hr)HPV-DNA [14] and the constitutive expression of the viral oncogenes E6 and E7 [15]. In an earlier study we showed by quantitative reverse-transcription-PCR that HPV16-E6-E7-mRNA is more sensitive and more specific than cytokeratin-(CK)19-mRNA for the detection of disseminated tumor cells in SLN [16]. The present study evaluates the prognostic value of hrHPV-E6-E7-mRNA in SLN biopsies of cervical cancer patients with pN0 status based on conventional histopathologic findings.

## RESULTS

### Patients enrolled

**Figure 1:**
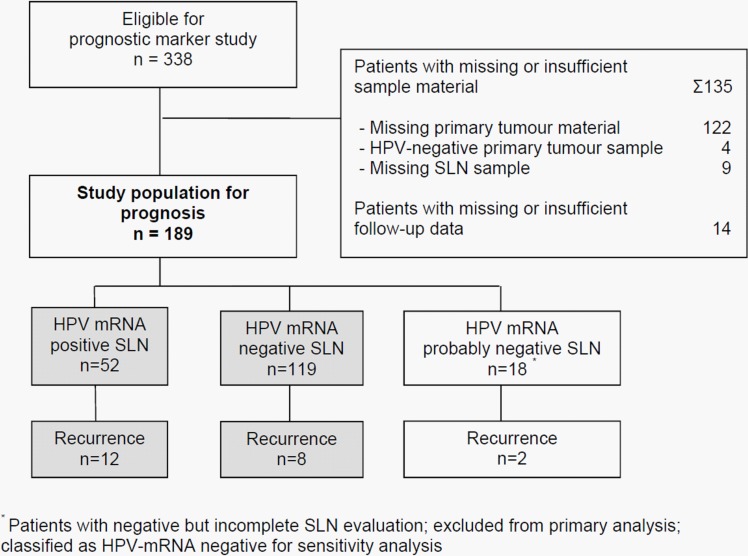
Study flow

We enrolled 189 out of 338 eligible patients (Figure 1). Median age was 40 years and FIGO stages ranged from IA1 to IIB. Further details are given in Table 1. Key data of patients excluded are shown in Table 2. Except for histology there were no remarkable differences for age, tumor size and grading. During the follow-up period disease recurred in 22 patients (Figure 1), overall 15 patients died (Supplementary Table S2). The probabilities of recurrence free survival and overall survival five years after surgery were 90.1% (95% CI 84.8 – 93.7%) and 93.3% (95% CI 88.2 - 96.2%), respectively.

**Table 1:** Characteristics of patients according to HPV mRNA status of sentinel lymph nodes (n=189)

	HPV mRNA status of sentinel lymph nodes			Total n = 189 (100.0 %)
	Positive n = 52 (27.5%)	Negative n = 119 (63.0%)	Probably negative n = 18 (9.5%)	
Age [years]				
Median	42.5	39.0	36.5	40.0
Range	22 - 76	23 - 77	26 - 61	22 - 77
BMI [kg/m^2^]*				
Median	24.8	23.8	22.9	24.0
Q1 – Q3	22.6 – 27.3	22.0 – 26.7	21.3 – 24.0	21.9 – 26.6
Histology, n (%)				
Squamous cell ca	41 (78.8)	97 (81.5)	14 (77.8)	152 (80.4)
Adenocarcinoma	7 (13.5)	19 (16.0)	3 (16.7)	29 (15.3)
Others	4 (7.7)	3 (2.5)	1 (5.6)	8 (4.2)
Tumor size [mm]*, n (%)				
≤ 20	17 (32.7)	75 (63.0)	10 (58.8)	102 (54.0)
>20 to 40	28 (53.8)	30 (25.2)	5 (29.4)	63 (33.3)
>40	7 (13.5)	14 (11.8)	2 (11.8)	23 (12.2)
Grading*, n (%)				
G1	5 (9.6)	11 (9.7)	0 (0.0)	16 (8.7)
G2	20 (38.5)	53 (46.9)	12 (66.7)	85 (46.4)
G3	27 (51.9)	49 (43.4)	6 (33.3)	82 (44.8)
HPV type primary tumor, n (%)				
16	25 (48.1)	74 (62.2)	12 (66.7)	111 (58.7)
18	16 (30.8)	23 (19.3)	2 (11.1)	41 (21.7)
Others	11 (21.2)	22 (18.5)	4 (22.2)	37 (19.6)

Abbreviations: BMI – body mass index, Q1 – first quartile, Q3 – third quartile, ca. – cancer

* Missing values BMI (n = 39), tumor size (n = 1), grading (n = 6)

**Table 2:** Characteristic of study population compared to patients excluded from study

	Study population (n = 189)	Patients not included (n = 149)	p-value
Age [years]			0.701*
Median	40	40	
Range	22 - 77	20 - 73	
Histology, n (%)			0.020**
Squamous cell cancer	152 (80.4)	101 (67.8)	
Adenocarcinoma	29 (15.3)	41 (27.5)	
Others	8 (4.2)	7 (4.7)	
Tumor size [mm], n (%)			0.718**
≤ 20	102 (54.3)	83 (56.1)	
>20 to 40	63 (33.5)	44 (29.7)	
>40	23 (12.3)	21 (14.2)	
Missing	1	1	
Grading, n (%)			0.045**
G1	16 (8.7)	4 (2.8)	
G2	85 (46.7)	79 (56.0)	
G3	82 (44.8)	58 (41.1)	
Missing	6	6	

* t-test.

** Chi^2^-test

### HPV status in primary tumors and sentinel lymph nodes

Close to 60% of the primary tumors were HPV16 positive, 22% were HPV18 positive and the remaining tumors were either HPV35-, HPV45- or HPV73-positive. HPV-mRNA of the respective HPV type could be detected by RT-nested-PCR in SLN of 52 patients (27.5%). 119 patients (63%) had negative and 18 patients (9.5%) had probably negative findings (Figure 1).

### Prognostic value of HPV-mRNA in sentinel lymph nodes

**Figure 2:**
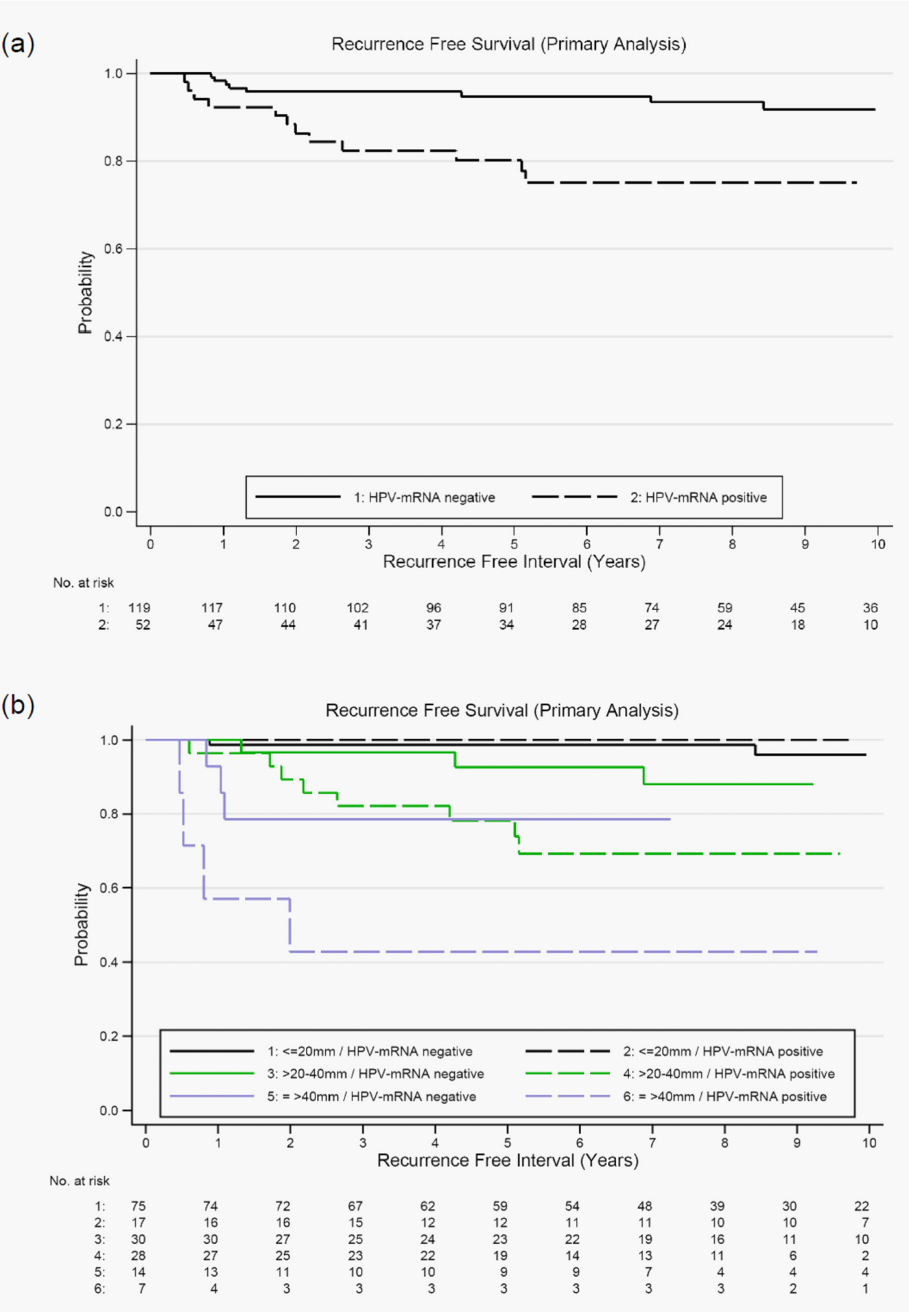
**a.** Kaplan-Meier curves of recurrence-free survival according to HPV-mRNA status of sentinel lymph nodes; log rank test *p* = 0.002 (primary analysis population *n* = 171) and **b.** Kaplan-Meier curves of recurrence-free survival according HPV-mRNA status of sentinel lymph nodes stratified by tumor size; log rank test for HPV-mRNA adjusted for tumor size *p* = 0.027 (primary analysis population *n* = 171).

In our primary analysis we included 171 patients with unequivocal HPV-mRNA findings as determined by RT-nested-PCR. Recurrence-free survival was significantly longer in patients with HPV-mRNA negative SLN (log rank *p* = 0.002). Five years after surgery 94.8% (95% CI 88.8 – 97.6%) of these patients survived without relapse compared to 80.2% (95% CI 66.2 – 88.8%) of patients with positive SLN (Figure 2a and Table 3). Results of the Cox-regression analysis are given in Table 4. The hazard ratio (HR) for disease recurrence was 3.8 (95% CI 1.5 – 9.3, *p* = 0.004) for HPV positive compared to patients with HPV-mRNA negative SNL. In single factor models tumor size was the most influential prognostic variable. Compared to patients with small tumors the risk of disease recurrence was 9-fold and 20-fold if tumor size was >20 to 40mm and >40mm, respectively. Grading and histology were not significantly associated with recurrence-free survival. The significant effect of HPV-mRNA in SLN was preserved in all two-factor Cox models. As expected from single factor models, the largest change in estimate was observed after inclusion of tumor size. HR for HPV positive compared to negative patients decreased to 2.8 (95% CI 1.1 – 7.0, *p* = 0.030). The independent prognostic values of tumor size and HPV-mRNA in SLN are shown in stratified Kaplan-Meier curves (Figure 2b). The benefit of HPV-mRNA as marker was obvious in patients with tumor size larger than 20mm due to the higher risk of recurrence (Table 3).

**Table 3:** Five-years rate of recurrence free survival according to HPV mRNA status of sentinel lymph nodes stratified by tumor size (primary analysis population, n=171)

Tumor size [mm]		HPV mRNA of SLN	Disease recurrence (n)	5-years rate of recurrence free survival (95% CI)
	all	all	20 (171)	90.3 (84.6 – 93.9) %
*	all	negative	8 (119)	94.8 (88.8 – 97.6) %
		positive	12 (52)	80.2 (66.2 – 88.8) %
**	≤ 20	negative	2 (75)	98.7 (90.9 – 99.8) %
		positive	0 (17)	100.0
>20 to 40		negative	3 (30)	92.6 (73.5 – 98.1) %
		positive	8 (28)	78.2 (57.8 – 89.6) %
>40		negative	3 (14)	78.6 (47.2 – 92.5) %
		positive	4 (7)	42.9 (9.8 – 73.4) %

Abbreviations: SLN – sentinel lymph node, CI – confidence interval

* Log rank test for HPV mRNA: p = 0.002

** Log rank test for HPV mRNA adjusted for tumor size: p = 0.027

**Table 4:** Strength of association between prognostic factors and recurrence free survival determined by Cox regression analysis (primary analysis population, n = 171)

		n	Hazard Ratio (95% CI)	p-value**
*Single factor models*				
HPV mRNA of SLN	negative	119	1	0.004
	positive	52	3.79 (1.54 – 9.27)	
Tumor size [mm]	≤ 20	92	1	<0.001
	>20 to 40	58	9.20 (2.03 – 41.53)	
	>40	21	19.68 (4.08 – 94.80)	
Histology	Squamous cell ca	138	1	0.869
	Adenocarcinoma	26	1.34 (0.44 – 4.03)	
	Others	7	1.21 (0.15 – 9.13)	
Grading*	G1	16	1	0.096
	G2	73	0.29 (0.06 – 1.29)	
	G3	76	0.96 (0.27 – 3.38)	
Age [years]		171	1.03 (1.00 – 1.07)	0.050
*Two factor models*				
HPV mRNA of SLN	negative	119	1	0.030
	positive	52	2.79 (1.10 – 7.03)	
Tumor size	≤ 20 mm	92	1	0.001
	>20 – 40 mm	58	6.66 (1.43 – 30.99)	
	>40 mm	21	17.77 (3.67 – 85.93)	
HPV mRNA of SLN	negative	119	1	0.004
	positive	52	3.81 (1.55 – 9.38)	
Histology	Squamous cell ca	138	1	0.877
	Adenocarcinoma	26	1.30 (0.43 – 3.92)	
	Others	7	0.85 (0.11 – 6.47)	
HPV mRNA of SLN*	negative	113	1	0.009
	positive	52	3.29 (1.34 – 8.08)	
Grading*	G1	16	1	0.142
	G2	73	0.32 (0.07 – 1.42)	
	G3	76	0.95 (0.27 – 3.35)	
HPV mRNA of SLN	negative	119	1	0.005
	positive	52	3.63 (1.48 – 8.89)	
Age [years]		171	1.03 (0.99 – 1.07)	0.070

Abbreviations: SLN – sentinel lymph node, CI – confidence interval, ca. – cancer

* Valid datasets: Grading and related two factor model n = 165

** Wald test

In a sensitivity analysis we included all 189 patients assigning those with probably negative HPV-mRNA status to the HPV-mRNA negative group. For HPV-mRNA positive compared to negative patients a crude HR for disease recurrence of 3.4 (95% CI 1.4 – 8.0, *p* = 0.004) and a tumor size adjusted HR of 2.5 (95% CI 1.0 – 6.0, *p* = 0.034) was estimated (Supplementary Table S4). For Kaplan-Meier curves of recurrent free survival and five-years of recurrence free survival see Supplementary Figure S1 and Supplementary Table S3.

Post-hoc we analyzed the effect of HPV-mRNA in SLN on total mortality. In the primary analysis population (*n* = 171) the crude and tumor size adjusted HR was 2.8 (95%CI 0.9 – 8.5, *p* = 0.060) and 2.5 (95%CI 0.8, 7.7, *p* = 0.113), respectively. The sensitivity analysis (*n* = 189) revealed a crude HR of 2.5 (95%CI 0.8 – 6.7, *p* = 0.084) which decreased after tumor size adjustment to 2.1 (95%CI 0.7 – 6.1, *p* = 0.154).

### Cancer-related recurrence and death according to HPV-mRNA status

Recurrence of disease was observed in 22 patients of whom 16 experienced loco-regional recurrence and 6 patients were diagnosed with distant metastasis outside the pelvis or in liver, lungs or spleen. The interval between primary treatment and recurrence varied between 5 and 101 months with late recurrence in 2 patients after 4 years, 2 patients after 5 years and 2 patients after 6 or 8 years, respectively. Overall 15 patients died during follow up. Interestingly, among the HPV-mRNA positive group 6 out of 7 deaths were directly related to cancer. In contrast, this was only the case for 3 out of 8 deaths among the HPV-mRNA negative group (Supplementary Table S2).

## DISCUSSION

Multiple studies have shown that SLN mapping in patients with early stage cervical cancer is feasible with excellent detection rates and sensitivity. Especially ultrastaging allows the detection not only of macro- and micrometastasis but also of ITC [6, 23]. However, whereas detection of micrometastasis may have a prognostic impact, ITC were shown not to be of prognostic value [6]. It is also of note that for breast cancer there is a lack of consensus in the literature about the predictive value of ITC as determined by cytokeratin staining in terms of both disease-free survival and death from disease [24]. It needs to be considered that cytokeratin stained cells do not invariably represent tumor cells. Indeed, a lack of specificity of cytokeratin-staining at a single cell level was shown by Bleiweiss and colleagues [13]. Thus, rather than using epithelial markers we had chosen HPV-E6-E7-mRNA as a molecular marker for the detection of tumor cells in SLN shown to be negative by conventional histopathology. HPV-DNA is less ideal as a marker because fragmented viral DNA may also be present in lymphocytes and would give rise to false-positive results [16].

This is the first multicenter prospective study which addresses the prognostic potential of HPV-mRNA in SLN of patients with pN0-status by systematic lymphadenectomy and conventional histopathology. We could show that recurrence-free survival was significantly longer for patients with HPV-mRNA negative SLN (log rank *p* = 0.002). By Cox-regression analysis the hazard ratio (95% confidence interval) for disease recurrence was 3.8 (1.5 – 9.3, *p* = 0.004) for HPV-mRNA positive compared to negative patients. Most importantly, after adjustment for tumor size as the most influential covariate for recurrence, the hazard ratio was still 2.8 (1.1 – 7.0, *p* = 0.030).

Although RT-nested-PCR is extremely sensitive it is not a quantitative assay. Therefore, in order to estimate the HPV16-mRNA levels in SLN, we also analyzed 77 SLN of a subset of patients by qRT-PCR. By this approach one tumor cell (SiHa) in a background of 10^5^ HPV-negative cells can be detected [16]. We could show that in SLN which were negative for micrometastasis by cytokeratin staining, one to 10^2^ HPV16-E6-E7 transcripts were detected in 50ng of total RNA (Supplementary Figure S2a). In terms of a tissue section this corresponds to single dispersed tumor cells. The agreement between the two different mRNA assays (qRT-PCR versus RT-nested-PCR) was high (kappa 0.8; Supplementary Figure S2b). As expected, the RT-nested-PCR approach was somewhat more sensitive.

There are several limitations of our study. First, a high percentage of eligible patients had to be excluded because of missing or insufficient sample material. To minimize the risk of contamination the study protocol required all samples to be taken as fresh frozen biopsies at the time of surgery. As shown in Table 2, patients excluded differed in histology (*p* = 0.02) and marginally in grading (*p* = 0.045). However, both characteristics did not influence recurrence-free survival in our study. Additionally, recurrence-free survival of the excluded subjects was similar at the regular end of the study (log rank test *p* = 0.42). Second, the power of our statistical analysis was lower than assumed. Moreover, the initial follow-up period of 3 years for recurrent disease was too short and had to be prolonged. The final number of 22 patients with recurrence was still lower than expected. Therefore the number of simultaneous covariates in the Cox-regression model had to be restricted and may have resulted in incomplete control of confounding. Third, the HPV-mRNA status of SLN was unclear in eighteen patients. For these cases the SLN count by the surgeon did not match with the number counted by the pathologist. The SLN available for HPV-mRNA analyses however were negative. When we included the data of patients with equivocal results, the sensitivity analysis revealed a weaker but still significant association (Supplementary Figure S1 and Supplementary Tables S3 and S4). This approach was specified in the statistical analysis plan before the recurrence status became known to the principal investigator. Forth, the biopsy taken from SLN for mRNA analysis may not have been representative of the whole node. Tumor cell distribution can be inhomogeneous in lymph nodes [25]. Thus, an unknown number of SLN may be false-negative.

In conclusion, our data suggest that the presence of HPV-E6-E7-mRNA in SLN of patients with pN0 status was associated with a significantly decreased recurrence-free survival. For the detection of isolated tumors cells HPV-E6-E7-mRNA as a biomarker is of unmatched specificity [16]. The clinical implications of HPV-mRNA in SLN and the net benefit for the patients with respect to therapy options will have to be addressed in further studies. It is envisaged that patients with IB1 tumors and HPV-mRNA negative SLN status can be spared from complete lymphadenectomy and be treated by locally-tailored surgery alone, whereas patients with HPV-mRNA positive SLN status may profit from surgery plus lymphadenectomy and adjuvant therapy.

## MATERIALS AND METHODS

### Study design

This study was performed as the longitudinal, prognostic part of a, to date, largest prospective, multi-center diagnostic study which addressed the SLN-concept in cervical cancer [4]. The study protocols of the diagnostic and the prognostic part were approved by the institutional review committee of the Jena University Hospital (0175-02/00). Between March 1999 and June 2008, 338 patients with cervical cancer were eligible for enrolment (Figure 1). All patients had to be followed for at least three years or were censored as case at an earlier time point. The data base was closed on October 29, 2009. At that time 189 patients with a median follow-up period of 3.1 (range 0.5 – 7.8) years fulfilled the study criteria. Due to the low rate of recurrent disease (*n* = 17) we decided to prolong the follow-up period. From July to November 2013 data on vital and recurrence status could be updated for 130 patients out of 172 who had no recurrence during the regular study period. Now, the median follow-up time was 8.1 (range 0.5 to 12.5) years. Here, we present results of our final analysis. Although initiated much earlier this study fulfils the criteria recommended for tumor marker prognostic studies (REMARK) published in 2005 [17].

### Patients

Inclusion criteria were all stages of invasive cervical cancer, intention of curative surgery and complete pelvic lymphadenectomy after removal of SLN (for details see [4]). Further inclusion criteria were R0 resection and pN0 status. Preoperatively detected metastatic disease, previous lymphadenectomy, tumor-involvement of the adnexae, or neoadjuvant therapy excluded from participation. All patients who signed informed consent were enrolled consecutively.

### Primary endpoint and prognostic variables

The primary endpoint was disease recurrence defined as local recurrence of primary disease, distant metastasis, death due to cervical cancer or death with unknown cause. In patients without an event observations were censored at date of last follow-up information that excluded disease recurrence. We primarily focused on the prognostic value of HPV-mRNA in pelvic SLN. HPV-mRNA-status was classified as positive, negative or probably negative. The last category was assigned to HPV-mRNA negative cases in which the SLN count by the surgeon did not match with the number counted by the pathologist. Tumor size (≤20mm, >20mm to 40mm, >40mm), histology, grading and, age (post-hoc due to prolonged follow-up) were considered as additional prognostic variables.

### Surgery and lymph node processing

Patients underwent complete pelvic lymphadenectomy either by laparoscopy or by open approach (for details see [4]). The pelvic area was inspected for stained/radioactive SLNs. SLNs were harvested first followed by systematic lymphadenectomy and tumor resection. Of each SLN a tissue section of 1mm thickness perpendicular to the long axis of the node was taken for mRNA analysis. To avoid contamination a fresh scalpel and forceps were taken for the dissection of each individual node. The biopsy was either immediately snap-frozen in liquid nitrogen or conserved in mRNA-later for storage at −20°C. All lymph nodes were processed identically by the pathologists. If metastatic disease was visible macroscopically, a single section was usually sufficient to confirm disease. Normal-appearing nodes were cut perpendicular to the long axis into 3- to 4mm sections and submitted for routine processing (no ultrastaging and no immunohistochemical staining). Indication for paraaortic lymphadenectomy varied between the different institutions and was given for tumor diameter larger than 3cm and/or vascular space involvement and/or deep cervical stroma infiltration and/or undifferentiated tumor (for details see [4]).

### HPV-genotyping of primary tumors

HPV-genotyping was performed using genomic DNA extracted from frozen biopsy material obtained from primary tumors. Thirty-seven different genital HPV types, including all hrHPV types can be amplified by the GP5+ and GP6+bio assay [18].

### Detection of HPV-mRNA in sentinel lymph nodes

#### (i) RNA extraction and cDNA synthesis

Approximately 30mg of tissue were homogenized (OMNI-TH homogenizer with dispersible tips) and total RNA was extracted thereof using the RNA Blood Mini Kit from Qiagen (Hilden, Germany) according to the manufacturer's instructions. DNase treatment was included. RNA concentration and quality were determined by spectrophotometry (NanoDrop ND-1000) and gel electrophoresis. One microgram of total RNA was reverse transcribed in a 20μl reaction comprising 20pmol CDS-primer (5′-T_n=30_VN-3′), 100nM of each dNTP, 50mM Tris-HCl (pH 8.3), 75mM KCl, 3mM MgCl_2_ 10mM DTT, 20U RNasin und 200U SuperScriptII (GibcoBRL). To allow optimal annealing, primer and RNA were incubated at 70°C for 10 min and cooled down on ice. The remaining reagents were then added in form of a master mix and cDNA synthesis was done at 42°C for one hour. The reaction was stopped at 70°C for 15 minutes and stored at −80°C until PCR analyses.

#### (ii) qualitative detection of viral transcripts by nested PCR (RT-nested-PCR)

For the detection of hrHPV-E6-E7 specific cDNA a nested-PCR protocol was used. By this approach one tumor cell (HPV16 positive cell line SiHa) can be detected in a background of 10^6^ HPV-negative cells. All primers are located in the E6 and E7 ORFs of the viral genome and detect spliced as well as unspliced early promoter-derived oncogene transcripts. Being polycistronic, these transcripts also encode the viral oncoprotein E7 [19]. Outer and inner primers of all 13 hrHPV types are given in Supplementary Table S1. The first PCR comprised 2μl of the cDNA reaction (equivalent to 100ng RNA), 50mM KCl, 10mM Tris-HCl (pH 8.3), 3.5mM MgCl_2_, 5% DMSO, 0.2mM of each dNTP, 20pmol of each primer und 1.25U AmpliTaq in an end volume of 50μl. After an initial denaturation step for 1 minute at 94°C, 40 cycles of 15 seconds at 94°C, 30 seconds at 54°C and 90 seconds at 72°C were performed. The final elongation step was prolonged by 4 minutes. For the second (nested) PCR, 2μl of the first reaction (1:3 dilution in H_2_O) was used. The reaction conditions were the same as for the first PCR except for the use of inner primers. All reactions were run in a Mastercycler Gradient (Eppendorf, Hamburg, Germany). PCR products were separated in 1.2% agarose gels for Southern blot analysis. Specificity was confirmed by hybridization using internal oligonucleotide probes (Supplementary Table S1).

To control for successful reverse transcription 1μl of cDNA (equivalent of 50ng total RNA) was taken for the amplification of GAPDH. The primers for GAPDH were: GGTGAAGGTCGGAGTCAACG (forward) and CAAAGTTGTCATGGATGACC (reverse). Reaction conditions were as for hrHPV-E6-E7 amplification but not in the nested format. Successful amplification was assessed in agarose gels.

#### (iii) quantitative detection of viral transcripts by real-time PCR

Beside RT-nested-PCR which provides a highly sensitive but only a qualitative readout, we also performed quantitative real-time analysis (qRT-PCR) for a subset of SLN (*n* = 77). This assay was described in detail by Häfner and colleagues (16). Target gene expression measured in duplicate was normalized to the geometric mean of the expression of the two most stable housekeeping genes in lymph nodes (HPRT, GAPDH).

### Statistical analysis

Sample size was calculated according to the rule of ten events per variable for proportional hazards regression analysis [20]. Beside a power calculation for detection of a specified size effect, the rule of ten events per variable is used in settings were the primary focus is directed at the estimation of the marker effect after adjustment for a set of standard variables as rationale for sample size [21]. We used this rule as a pragmatic sample size justification for the following reasons: First, our prognostic study was performed in a subgroup of patients participating in our prospective, multicenter diagnostic study which addressed the sentinel lymph node concept in cervical cancer. The total sample size, which was previously calculated according to the objective of the diagnostic study, restricted the number of eligible patients for the prognostic part. Second, due to the early phase of marker evaluation we focused on estimating the independent marker effect after adjustment for a set of pre-defined variables rather than significance testing. Taking into account five independent variables 50 patients with recurrence and 334 overall would be required for analysis assuming a recurrence rate of 15% within three years. Median follow-up time was calculated by the reverse Kaplan-Meier method [22]. Kaplan-Meier curves were generated to display recurrence-free survival and to estimate 5-years rates with 95% confidence intervals (CI). Strength of association between prognostic variables and recurrence-free survival was quantified by crude and adjusted hazard ratios (HR) from Cox regression models with 95% CI. The population for the primary analyses comprised only patients with unequivocal HPV-mRNA findings. For sensitivity analyses we assigned patients with probably negative HPV-mRNA status to the HPV-mRNA-negative group. We analyzed overall survival as a post-hoc specified endpoint in a similar way. Statistical comparisons were made by t-test, Chi^2^ test, log rank test and Wald test as appropriate. For comparison of laboratory methods the kappa statistic was calculated. The two-sided level of significance was 0.05. All data were managed in an ACCESS database and were analyzed with statistical software SAS 9.3 and Stata 12.0.

## SUPPLEMENTARY MATERIAL FIGURES AND TABLES


