# Supplementary material for: Prognostic value of HPV-mRNA in sentinel lymph nodes of cervical cancer patients with pN0-status
Source: Oncotarget. 2015 May 14;6(26):23015–25. doi: 10.18632/oncotarget.4132 (PMC4673218; doi:10.18632/oncotarget.4132)
Supplement: Supplementary file 1 [file oncotarget-06-23015-s001.pdf]

Prognostic value of HPV-mRNA in sentinel lymph nodes of cervical cancer patients with pN0-status

Supplementary Material

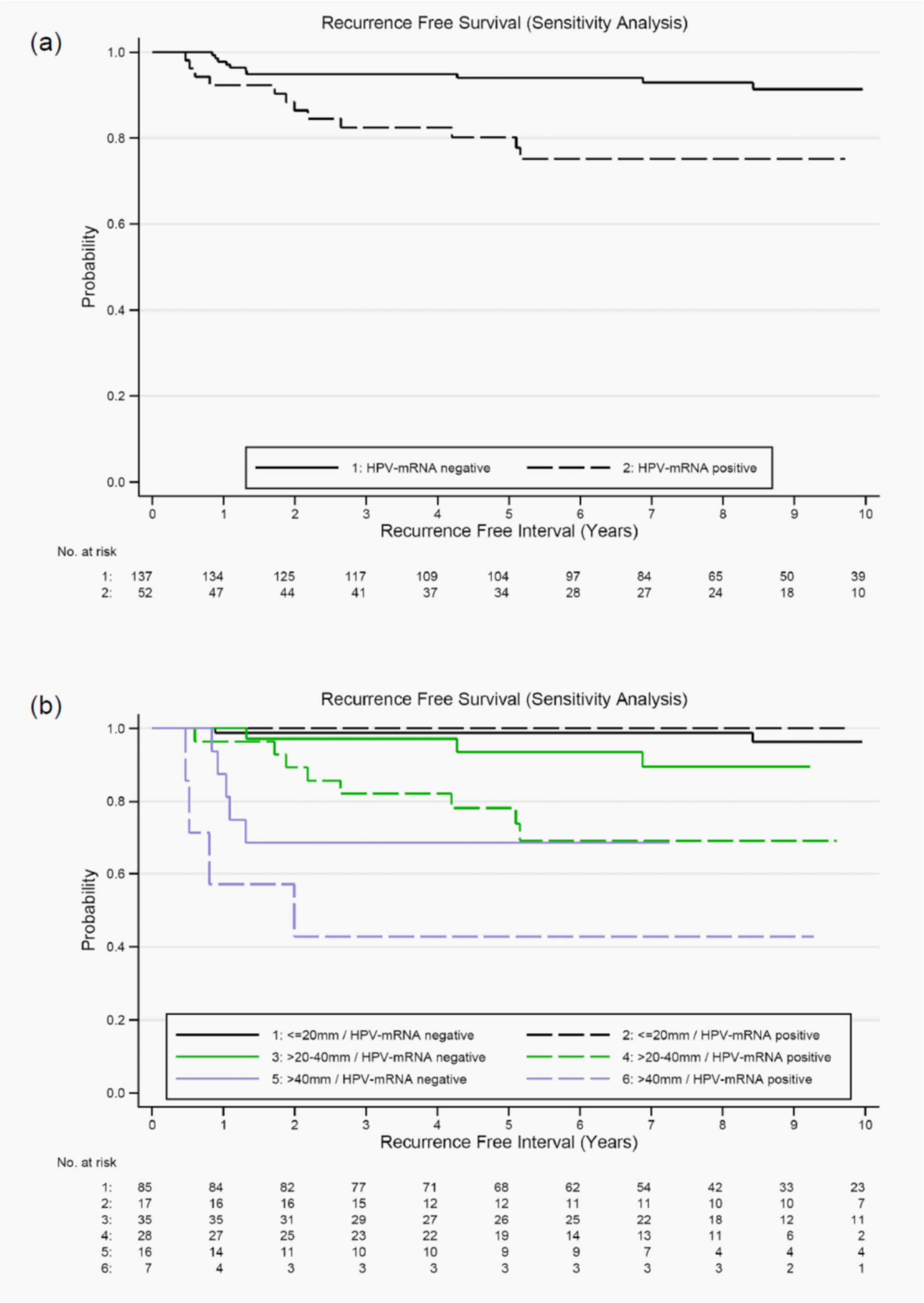

Figure S1: (a) Sensitivity analysis - Kaplan Meier curves of recurrence-free survival according to HPV-mRNA status of sentinel lymph nodes; log rank test p=0.002 (study population n=189) and (b) Sensitivity analysis - Kaplan Meier curves of recurrence-free survival according to HPV-mRNA status of sentinel lymph nodes stratified by tumor size; log rank test for HPV-mRNA adjusted for tumor size p=0.028 (study population n=189).

(a)

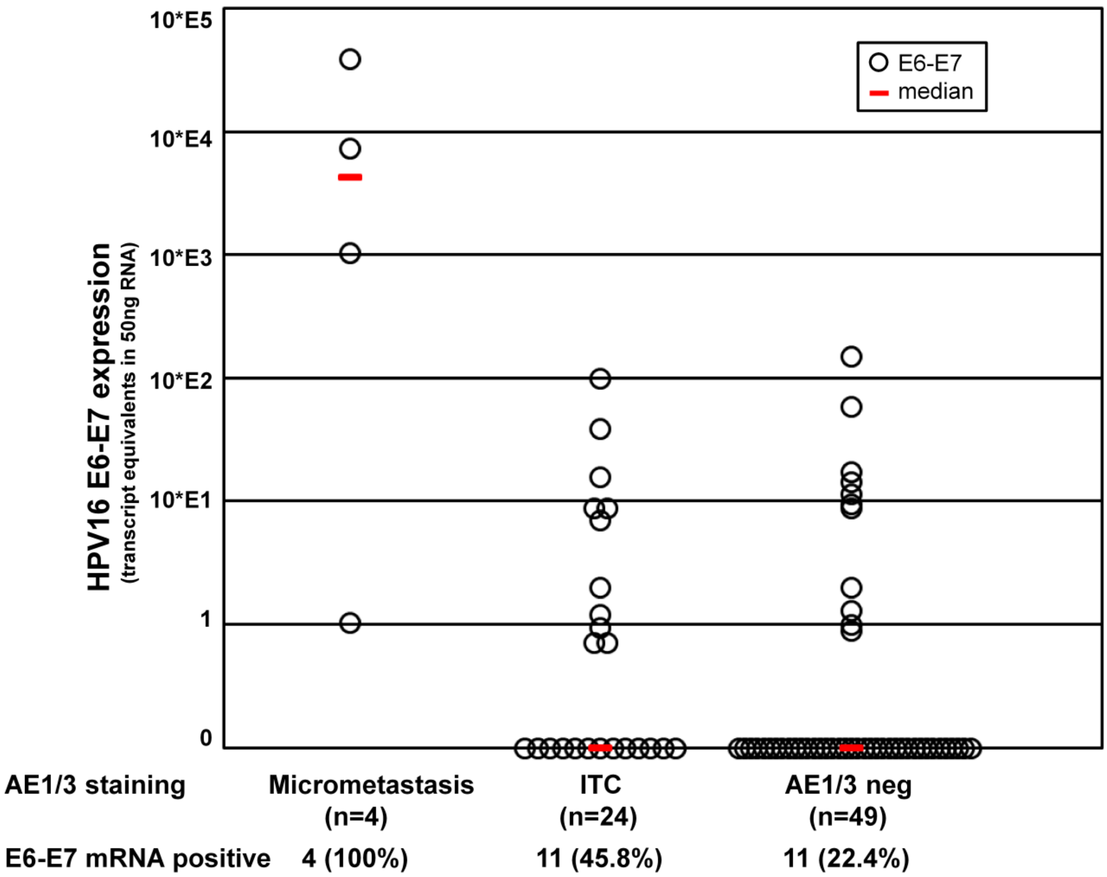

(b)

		nested PCR +	nested PCR -	
qPCR	+	24	2	26
	-	5	46	51
		29	48	77

kappa = 0.802

Figure S2: HPV16 E6-E7-mRNA expression as determined by qRT-PCR and RT-nested-PCR in relation to immunohistochemical staining for cytokeratins (AE1/3) in 77 SLN scored negative by conventional histopathology. (a) Expression levels (qRT-PCR) corresponding to HPV16-E6-E7 transcripts in 50ng of total RNA (see Häfner et al. [16]) are shown on the y-axis. 50ng of total RNA corresponds to $5x10^3$ cells. On the x-axis the results of AE1/3 staining and quantitative RT-nested-PCR are shown. ITC refers to single dispersed tumor cells. (b) Overall agreement between qRT-PCR and RT-nested PCR.

Supplementary Table S1: Primers for the detection of hrHPV-E6-mRNA by RT-nested-PCR and oligonucleotide probes for Southern blots

HPV type	1st PCR			2nd PCR			Southern Blot
	forward	reverse	length FLT (E6*I) [bp]	forward	reverse	length FLT (E6*I) [bp]	hybridisation probe
**16**	ACAGGAGCGACCCAGAAAGTTACC	ATGGTTTCTGAGAACAGATGGGGC	739 (556)	TGGGGCACACAATTCCTAGTGTGC	AGTTACCACAGTTATGCACAGAGC	704 (521)	TCATGCAATGTAGGTGTATCTCC
**18**	ACAAGCTACCTGATCTGTGC	CTGAGTCGCTTAATTGCTCG	554 (373)	GGAACTGAACACTTCACTGC	TGACATAGAAGGTCAACCGG	512 (331)	GCAGCATGCGGTATACTGTCTC
**31**	AATCCTGCAGAAAGACCTCGG	GGCACACGATTCCAAATGAGC	717 (514)	CGGAAATTGCATGAACTAAGC	CCAAATGAGCCCATTAACAGC	688 (485)	ACTCCGTGTGGTGTGTCGTC
**33**	TGCATGATTTGTGCCAAG	TTTGTACCTTCAGGATCG	759 (481)	CCAAGCATTGGAGACAAC	GATCGGCCATTGTAGATG	733 (455)	CACTAATTTTAGATAAGAACCGCAAA
**35**	TCAGGACCCAGCTGAACGACC	TATGCTCTCTGTGAACAGCCG	746 (563)	GAACGACCTTACAAACTGCATG	CACACTATTCCAAATGTGCCC	704 (521)	GGTCAGTTGCCTCGGGTTCC
**39**	TCACAATCCTGCAGAACGGC	ACAATTTCCTGCAAGGTGGG	509 (326)	TGCCAGACCTGTGCACAACG	AGGTGGGCTTTGGTCCACGC	467 (284)	GGTCGTCTGCAATAGACACAGG
**45**	ATGGCGCGCTTTGACGATCC	CTGACTCGCTTAATTGCTCG	591 (406)	GCAACGACCCTACAAGCTAC	CATTCTGAGGTTCCAAATGC	520 (335)	TACCTCTGTGCGTTCCAATGTT
**51**	GGGAAAGACCACGAACGCTGC	CTAGTTGTTCGCACAACACGG	753 (528)	GCTGCATGAATTATGTGAAGC	CAAACCAGGCTTAGTTCGCCC	716 (491)	GCGAAGGGTGTCTCCACTGC
**52**	CCAGCAACACGACCCCGGACC	AATGTGCCCAACAGCATTTGC	705 (450)	CCCTGCACGAATTGTGTGAGG	TAGAGTACGAAGGTCCGTCGC	660 (405)	TGTCTCCACGCATGACGTTACA
**53**	CATCCATGGATCGTCAGTTATTTG	TTGCAGTTATCGCCTTGTTGC	790 (607)	AAGAGCGACCACGTACATTGC	CACTGTGCCCATAAGCATTTG	715 (532)	GAGGCTAGCCCCGTACACTGAAC
**56**	CAACAATCCACAGGAACGTCC	GCTTGCTGTGGCCGCTCCTGC	605 (350)	GCCTGCACCACTTGAGTGAGG	TCCTCATCCTCTGAGCTGTCC	541 (286)	GTTTGTCTCCAGCACCCCAAAC
**58**	ATTTGTGTCAGGCGTTGG	CCGTTTGTACCTTCAGGG	759 (481)	CGTTGGAGACATCTGTGC	CAGGGTCATCCATTGCAG	734 (456)	ACTCACTTATTTTAGATAGCAATCGTAAG
**59**	TGAGGATCCTACACAACGACC	AAACAGCTGCTGTAAGGCTCG	752 (353)	ACAACGACCATACAAACTGCC	CTCGCAATCCGTCTTGCGAGC	727 (328)	TCTCTTTCTTGCAGTTCCCCTT
**66**	CATATTCAGCAATACACAGG	TCAATTTCCGTTTGCGGTGC	527 (272)	ACGTCCACGAAGCCTGCACC	TGCAACGTTGGTACTTTACC	467 (212)	TCCAACACTGCAAACATGACCC
**68**	AGTTGTCTATACCAATGGCGC	GGTTTCCGTTGCACACCACGG	825 (642)	CGCTATTTCACAACCCTGAGT	AGCAGTTCTACGTTCCGCAGG	764 (581)	GACGCTGTTGTTCGTCCCGTC
**73**	GACTGTGTGTTTTGCCAACG	AGGTCAATTTCGGTTGTTGG	429 (246)	TGATTTTGCATTTAGTGATTTGTT	CACCACAGTTGCAGATGGTC	313 (130)	GCCATATACTGATTGTCTATATCGCCT
**82**	CATAAGAGAAAGACCACGAACG	GGTGTTAACTCCAACACTATGTCC	502 (277)	TGTGAAGCCTGCAATACGTC	TACCACGCATGGCGTTATTA	428 (203)	TTCTTCAGGCCCAAGTGGTCTC

The RT-nested-PCR assay for hrHPV-E6-mRNA allows the detection of solely matured spliced (E6*I) and un-spliced (E6-full length) viral oncogene transcripts which can only be found in intact tumor cells

Supplementary Table S2: Frequency of adjuvant therapy, para-aortic lymph node dissection and death in relation to HPV mRNA status

	HPV mRNA status of sentinel lymph nodes			
	Positive	Negative	Probably negative	Total
**Adjuvant therapy post OP**	19 / 52 (36.5%)	26 / 119 (21.8%)	7 / 17 (41.2%)	52 / 188 (27.7%)
**Para-aortic lymph node dissection**	16 / 52 (30.8%)	28 / 119 (23.5%)	7 / 18 (38.9)	51 / 189 (27.0%)
**Death* (due to cervical cancer)**	7 (6) / 52	6 (2) / 119	2 (1) / 18	15 (9) / 189

* without percentage due to different duration of observation

Supplementary Table S3: Five-years rate of recurrence free survival according to HPV mRNA status of sentinel lymph nodes stratified by tumor size (sensitivity analysis population, n=189)

Tumor size [mm]		HPV mRNA of SLN	Disease recurrence (n)	5-years rate of recurrence free survival (95% CI)
	all	all	22 (189)	90.1 (84.8 – 93.7) %
**	all	negative*	10 (137)	94.0 (88.4 – 97.0) %
		positive	12 (52)	80.2 (66.2 – 88.8) %
***	≤ 20	negative*	2 (85)	98.8 (91.9 – 99.8) %
		positive	0 (17)	100.0 %
	>20 to 40	negative*	3 (35)	93.5 (76.3 – 98.4) %
		positive	8 (28)	78.2 (57.8 – 89.6) %
	>40	negative*	5 (16)	68.8 (40.5 – 85.6) %
		positive	4 (7)	42.9 (9.8 – 73.4) %

Abbreviations: SLN – sentinel lymph node, CI – confidence interval

* Probably or definitely negative

** Log rank test for HPV mRNA: p=0.002

*** Log rank test for HPV mRNA adjusted for tumor size: p=0.028

Supplementary Table S4: Strength of association between prognostic factors and recurrence free survival determined by Cox regression analysis (sensitivity analysis population, n=189)

		n	Hazard Ratio (95% CI)	p-value***
Single factor models				
HPV mRNA of SLN	probably or definitely negative	137	1	0.004
	positive	52	3.45 (1.48 – 7.98)	
Tumor size [mm]**	≤ 20	102	1	<0.001
	>20 to 40	63	9.43 (2.08 – 42.54)	
	>40	23	27.01 (5.82 – 125.19)	
Histology	Squamous cell ca.	152	1	0.487
	Adenocarcinoma	29	1.28 (0.42 – 3.82)	
	Others	8	2.41 (0.55 – 10.47)	
Grading**	G1	16	1	0.194
	G2	85	0.38 (0.09 – 1.52)	
	G3	82	0.88 (0.25 – 3.08)	
Age [years]		189	1.03 (0.99 – 1.06)	0.074
Two factor models				
HPV-mRNA of SLN**	negative*	136	1	0.034
	positive	52	2.54 (1.07 – 6.03)	
Tumor size**	≤ 20 mm	102	1	<0.001
	>20 – 40 mm	63	7.09 (1.53 – 32.87)	
	>40 mm	23	24.42 (5.24 – 113.60)	
HPV-mRNA of SLN	negative*	137	1	0.006
	positive	52	3.32 (1.41 – 7.75)	
Histology	Squamous cell ca.	152	1	0.747
	Adenocarcinoma	29	1.24 (0.41 – 3.71)	
	Others	8	1.72 (0.38 – 7.59)	
HPV-mRNA of SLN**	negative*	131	1	0.010
	positive	52	3.05 (1.31 – 7.10)	
Grading**	G1	16	1	0.291
	G2	85	0.43 (0.10 – 1.74)	
	G3	82	0.90 (0.25 – 3.16)	
HPV mRNA of SLN	negative*	137	1	0.005
	positive	52	3.30 (1.42 – 7.64)	
Age [years]		189	1.03 (0.99 – 1.06)	0.106

Abbreviations: SLN – sentinel lymph node, CI – confidence interval, ca. – cancer
* Probably or definitely negative
** Valid datasets: Tumor size and related two factor model n=188, grading and related two factor model n=183
*** Wald test
